# An insight into the isolation, enumeration, and molecular detection of *Listeria monocytogenes* in food

**DOI:** 10.3389/fmicb.2015.01227

**Published:** 2015-11-03

**Authors:** Jodi Woan-Fei Law, Nurul-Syakima Ab Mutalib, Kok-Gan Chan, Learn-Han Lee

**Affiliations:** ^1^Biomedical Research Laboratory, Jeffrey Cheah School of Medicine and Health Sciences, Monash UniversityBandar Sunway, Malaysia; ^2^UKM Medical Molecular Biology Institute, UKM Medical CentreKuala Lumpur, Malaysia; ^3^Division of Genetics and Molecular Biology, Institute of Biological Sciences, Faculty of Science, University of MalayaKuala Lumpur, Malaysia

**Keywords:** *Listeria* species, isolation, enumeration, molecular detection, foodborne pathogens

## Abstract

*Listeria monocytogenes*, a foodborne pathogen that can cause listeriosis through the consumption of food contaminated with this pathogen. The ability of *L. monocytogenes* to survive in extreme conditions and cause food contaminations have become a major concern. Hence, routine microbiological food testing is necessary to prevent food contamination and outbreaks of foodborne illness. This review provides insight into the methods for cultural detection, enumeration, and molecular identification of *L. monocytogenes* in various food samples. There are a number of enrichment and plating media that can be used for the isolation of *L. monocytogenes* from food samples. Enrichment media such as buffered *Listeria* enrichment broth, Fraser broth, and University of Vermont Medium (UVM) *Listeria* enrichment broth are recommended by regulatory agencies such as Food and Drug Administration-bacteriological and analytical method (FDA-BAM), US Department of Agriculture-Food and Safety (USDA-FSIS), and International Organization for Standardization (ISO). Many plating media are available for the isolation of *L. monocytogenes*, for instance, polymyxin acriflavin lithium-chloride ceftazidime aesculin mannitol, Oxford, and other chromogenic media. Besides, reference methods like FDA-BAM, ISO 11290 method, and USDA-FSIS method are usually applied for the cultural detection or enumeration of *L. monocytogenes*. most probable number technique is applied for the enumeration of *L. monocytogenes* in the case of low level contamination. Molecular methods including polymerase chain reaction, multiplex polymerase chain reaction, real-time/quantitative polymerase chain reaction, nucleic acid sequence-based amplification, loop-mediated isothermal amplification, DNA microarray, and next generation sequencing technology for the detection and identification of *L. monocytogenes* are discussed in this review. Overall, molecular methods are rapid, sensitive, specific, time- and labor-saving. In future, there are chances for the development of new techniques for the detection and identification of foodborne with improved features.

## Introduction

*Listeria monocytogenes* has become an important foodborne pathogen and it can be found in a variety of foods which include raw foods and processed foods (Gasanov et al., 2005; Janzten et al., 2006; Liu, 2006; Jeyaletchumi et al., 2010a; Välimaa et al., 2015). *L. monocytogenes* has been a serious threat to the food industry due to its ability to survive the most common food processing conditions such as extreme pH, high salt concentration, low water activity, and refrigeration temperatures (Liu, 2006; Zunabovic et al., 2011; Jadhav et al., 2012). It is known that *L. monocytogenes* can be eliminated or reduced by pasteurization process because it cannot survive pasteurization temperatures in food processing (Jadhav et al., 2012). For instance, a study conducted by Murphy et al. (2003) showed that hot water pasteurization and steam pasteurization resulted in a 7 log_10_ (CFU/g) reduction of *L. monocytogenes* in inoculated single packaged fully cooked chicken breast fillets, 227 g packaged fully cooked chicken strips and 454 g packaged fully cooked chicken strips when pasteurized at 90°C for 5, 25, and 35 min, respectively. However, post-processing or post-pasteurization contamination by *L. monocytogenes* may occur due to cross-contamination or biofilms (Jadhav et al., 2012). *L. monocytogenes* can attach to food contact surfaces such as stainless steel and polystyrene during food processing and form biofilms which is important for their survival in hostile environments (Jadhav et al., 2012; Da Silva and De Martinis, 2013; Välimaa et al., 2015). Biofilms may persist for a long period of time and they can tolerate high concentrations of disinfectants, sanitizers, and antimicrobials (Välimaa et al., 2015). Hence, this may result in the contamination of food contact surfaces which then lead to higher risk of food contamination during and/or after processing. Food contaminated with *L. monocytogenes* has posed a great concern to the food industry as it can cause serious infection known as listeriosis when ingested and it is also one of the causes of recalls which may result in large economic losses (Jemmi and Stephan, 2006). Thus, microbiological food testing is important to ensure the safety of food products (Dwivedi and Jaykus, 2011; Välimaa et al., 2015).

## Detection and Identification of *Listeria monocytogenes*

### Enrichment Media and Selectivity

There are various selective enrichment and plating media that have been developed and utilized for the isolation and detection of *L. monocytogenes* in food and environmental samples. As required by majority of the regulatory agencies, the isolation methods must be able to detect one *Listeria* organism per 25 g of food. In order to achieve this sensitivity, enrichment methods are required to allow the organism to grow and reach a detectable level of ∼10^4^–10^5^ CFU ml^-1^, before plating onto selective media and confirmation of cultures. Antimicrobial agents are employed in enrichment and plating media to suppress competing microflora as *Listeria* cells are slow growing and can be rapidly out-grown by competitors. The most common selective agents are acriflavine, nalidixic acid, and cycloheximide (Beumer and Hazeleger, 2003; Gasanov et al., 2005; Janzten et al., 2006; Jeyaletchumi et al., 2010a). The function of acriflavine is to inhibit the growth of other Gram-positive bacteria and it is often used in combination with other selective agents, for instance, polymyxin B-sulfate, cycloheximide, potassium thiocyanate, and nalidixic acid. Nalidixic acid is used for the inhibition of Gram-negative bacteria while cycloheximide is used for the inhibition of fungi (Beumer and Hazeleger, 2003; Janzten et al., 2006; Jeyaletchumi et al., 2010a). Besides, there are other antimicrobials that are usually added into the media such as broad-spectrum ceftazidime and moxalactam as well as lithium chloride (Janzten et al., 2006).

In addition, esculin is an important carbohydrate that is usually incorporated in *Listeria* enrichment and plating media (Bush and Donnelly, 1992; Janzten et al., 2006; Jeyaletchumi et al., 2010a). All *Listeria* sp. are capable of esculin hydrolysis and this process will result in the formation of an intense black color in the media. This is due to the presence of esculin and ferric iron in the media, in which the ferric iron forms complex with 6,7-dihidroxycoumarin, the product of esculin cleavage by β-D-glucosidase, resulting in a black precipitate (Fraser and Sperber, 1988; Janzten et al., 2006; Jeyaletchumi et al., 2010a). Hence, cultures that produce an intense black color indicate the presence of *Listeria*.

The regulatory agencies have recommended several selective enrichment media for *L. monocytogenes* such as buffered *Listeria* enrichment broth (BLEB), Fraser broth, and University of Vermont Medium (UVM) *Listeria* enrichment broth. BLEB is recommended in the US Food and Drug Administration (FDA) bacteriological and analytical method (BAM) for the isolation and identification of *L. monocytogenes*. BLEB is a modification of the formula by Lovett et al. (1987), in which disodium phosphate is added into the medium to increase the buffering capacity of the medium, thus, resulting in the improvement of enrichment properties. Selective agents which include acriflavine, cycloheximide, and nalidixic acid are added into the medium after an initial 4 h of non-selective pre-enrichment (Magalhães et al., 2014).

University of Vermont Medium *Listeria* enrichment broth is recommended in the US Department of Agriculture-Food and Safety (USDA-FSIS) method for the isolation and detection of *L. monocytogenes*. UVM is based on the formulation of Donnelly and Baigent (1986). This enrichment broth is suggested as the primary enrichment broth for the recovery of heat-injured *L. monocytogenes*. Nalidixic acid and acriflavine are the selective agents added into the medium (Magalhães et al., 2014).

International Organization for Standardization (ISO) 11290 method has suggested the use of Fraser broth for the selective enrichment of *L. monocytogenes* in food and environmental samples. Fraser broth is based on the formulation of Fraser and Sperber (1988) whereby it is a modification of USDA secondary enrichment broth by the addition of ferric ammonium citrate and lithium chloride. The selective agents added into Fraser broth are nalidixic acid and acriflavine (Magalhães et al., 2014). Presumptive *L. monocytogenes* can be detected within 48 h by using Fraser broth (Fraser and Sperber, 1988).

The frequently recommended selective differential plating media by FDA-BAM, ISO, and USDA for the isolation of *Listeria* sp. are PALCAM (polymyxin acriflavin lithium-chloride ceftazidime aesculin mannitol) and Oxford (Zunabovic et al., 2011). PALCAM and Oxford are both useful for the isolation of *Listeria* sp. from food samples with injured *Listeria* cells and/or rich in competitive microflora (El Marrakchi et al., 2005). PALCAM agar was initially developed by Van Netten et al. (1989) for the detection and enumeration of *L. monocytogenes* and other *Listeria* sp. from food samples. PALCAM agar consists of Columbia Blood Agar with 23.0 g/L protease peptones, 0.5 g/L glucose, 1.0 g/L starch, 3.0 g/L yeast extract, and 5.0 g/L sodium chloride. The selectivity of this medium is achieved by the addition of 15 g/L lithium chloride, 0.01 g/L polymyxin B, 0.005 g/L acriflavine, and 0.02 g/L ceftazidime. The differentiation on PALCAM agar is based on (1) esculin hydrolysis, addition of 0.8 g/L esculin and 0.5 g/L ferric salt and (2) mannitol fermentation, addition of 10 g/L mannitol and 0.08 g/L phenol red (Van Netten et al., 1989; Magalhães et al., 2014). PALCAM agar plated with bacteria is usually incubated for 24–48 h at 37°C (Jamali et al., 2013; Ajay Kumar et al., 2014; Osman et al., 2014). Since all *Listeria* sp. can hydrolyze esculin, they are visually confirmed by a blackening of the medium and their colonies are about 2 mm diameter, gray-green in color with a black sunken center and a black halo. Occasionally, *Enterococcus* sp. or *Staphylococcus* sp. may grow on PALCAM agar. However, they can be distinguished from *Listeria* sp. via mannitol fermentation. Mannitol fermentation causes a color change in the colony and/or surrounding medium from gray or red to yellow due to the production of acids. Colonies of these mannitol fermenting organisms are yellow with a yellow halo or gray with a brown-green halo (Van Netten et al., 1989; Ajay Kumar et al., 2014; Osman et al., 2014).

Oxford agar was initially developed by Curtis et al. (1989) for the isolation of *L. monocytogenes* from clinical specimens. Oxford agar has been extensively used in many studies for the isolation and detection of *L. monocytogenes* from various food samples (Pinto et al., 2001; Rudol and Scherer, 2001; Gudbjörnsdóttir et al., 2004; Mena et al., 2004; Alessandria et al., 2010). Oxford consists of Columbia Blood Agar with 23 g/L protease peptones, 5.0 g/L sodium chloride, and 1.0 g/L starch. The selectivity of Oxford is achieved by the addition of 15 g/L lithium chloride, 0.005 g/L acriflavine, 0.02 g/L colistin sulfate, 0.4 g/L cycloheximide, 0.002 g/L cefotetan, and 0.01 g/L fosfomycin. The differentiation of *Listeria* sp. on Oxford agar is based on esculin hydrolysis which is aided by the addition of 1 g/L esculin and 0.5 g/L ferric ammonium citrate into the agar (Curtis et al., 1989; Janzten et al., 2006; Magalhães et al., 2014). Oxford agar is incubated for 24–48 h at 37°C after plating (Curtis et al., 1989; Alessandria et al., 2010). After 24 h of incubation, *L. monocytogenes* colonies are olive-green with a black halo. After 48 h of incubation, *L. monocytogenes* colonies are about 2–3 mm in diameter, the color turns darker with a black sunken center and surrounded by black zones. Other *Listeria* sp. colonies have a similar appearance to *L. monocytogenes* colonies. Colonies of other *Listeria* sp. are black with a black halo after 24 h of incubation and they remain the same after 48 h of incubation but with a sunken center (Curtis et al., 1989; Magalhães et al., 2014). *Staphylococcus* sp. may grow on Oxford agar occasionally and their colonies are yellow in color, irregular in size as well as shape (Curtis et al., 1989). A variation of Oxford agar has been developed and it is known as Modified Oxford Agar (MOX). MOX is recommended for the isolation and identification of *L. monocytogenes* from processed meat and poultry products whereas Oxford agar is recommended for the isolation of *L. monocytogenes* from enrichment broth cultures (Magalhães et al., 2014).

The main limitation of PALCAM and Oxford is the inability to distinguish between *L. monocytogenes* from non-pathogenic *Listeria* sp. (El Marrakchi et al., 2005; Zunabovic et al., 2011). Hence, these plating media are not able to provide a rapid detection of *L. monocytogenes* from foods. This has led to the development of chromogenic media which can improve the isolation of *L. monocytogenes* as they are able to differentiate *L. monocytogenes* and/or pathogenic *Listeria* sp. from other non-pathogenic *Listeria* sp. (Beumer and Hazeleger, 2003). Chromogenic media detect essential determinants of pathogenicity of *Listeria* sp. and majority of these media are commercially available as ready-to-use plates (Janzten et al., 2006; Zunabovic et al., 2011). Besides, presumptive *L. monocytogenes* can be identified after 24 h by using chromogenic media (Jeyaletchumi et al., 2010a). Several studies have demonstrated that chromogenic media such as Agar *Listeria* according to Ottaviani and Agosti (ALOA) and CHROMagar^TM^ *Listeria* are more sensitive, specific, time and cost saving in *L. monocytogenes* detection compared to non-chromogenic media such as PALCAM and Oxford (Vlaemynck et al., 2000; Hegde et al., 2007; Jamali et al., 2013).

Agar *Listeria* according to Ottaviani and Agosti is a chromogenic medium developed by Ottaviani et al. (1997) for the isolation of *Listeria* sp. and specific detection of *L. monocytogenes*. The selectivity of ALOA is achieved by the addition of lithium chloride and antimicrobials such as ceftazidime, polymyxin B, nalidixic acid, and cycloheximide (Beumer and Hazeleger, 2003; Magalhães et al., 2014). The differentiation of *Listeria* sp. on ALOA is achieved by the incorporation of chromogenic substrate (5-bromo-4-chloro-3-indolyl-β-D-glucopyranoside, X-glucoside) in the medium for the detection of β-D-glucosidase activity, common to all *Listeria* sp. The differentiation of *L. monocytogenes* from other *Listeria* sp. is based on the production of phosphatidylinositol-specific phospholipase C (PI-PLC) which is encoded by the virulence gene *plc*A present in *L. monocytogenes*. ALOA detects PI-PLC that is present in *L. monocytogenes* and in some strains of *L. ivanovii* through the hydrolysis of L-α-phosphatidylinositol in the medium by PI-PLC. This results in the production of water insoluble fatty acids and the formation of an opaque halo around the colonies. In ALOA, all *Listeria* sp. produce blue-green colonies and pathogenic *Listeria* sp. such as *L. monocytogenes* and *L. ivanovii* produce blue-green colonies with an opaque halo (Ottaviani et al., 1997; Beumer and Hazeleger, 2003; Janzten et al., 2006; Jeyaletchumi et al., 2010a; Zunabovic et al., 2011; Magalhães et al., 2014; Park et al., 2014).

CHROMagar^TM^ *Listeria* (Becton Dickson Diagnostics) is one of the variations of ALOA that have been developed for the isolation and detection of *L. monocytogenes*. On CHROMagar^TM^ *Listeria*, colonies of *L. monocytogenes* are blue with a white halo and colonies of other *Listeria* sp. are blue without halo. Some strains of *L. ivanovii* may grow on CHROMagar^TM^ *Listeria* and they also produce blue colonies with a white halo (Magalhães et al., 2014). Additional variations of ALOA and commercially available media include Biosynth Chromogenic Medium (BCM) *L. monocytogenes* detection system (Biosynth), Compass L. mono (Biokar Diagnostics), Brilliance^TM^ *Listeria* Agar (Oxoid) and chromID Ottaviani Agosti Agar (bioMérieux; Janzten et al., 2006; Zunabovic et al., 2011).

Rapid’ *L. mono* agar is a chromogenic medium that operates differently than ALOA. Rapid’ *L. mono* agar detects PI-PLC that is present in *L. monocytogenes* and *L. ivanovii* through the hydrolysis of a different substrate by PI-PLC, which is 5-bromo-4-chloro-3-indolyl-myo-inositol-1-phospjhate (X-IP). Cleavage of X-IP by PI-PLC results in the production of blue colonies. Hence, *L. monocytogenes* and *L. ivanovii* appear as blue colonies on Rapid’ *L. mono* agar. Furthermore, the addition of xylose into the medium enables the differentiation of *L. monocytogenes* from *L. ivanovii*. The ability of *L. ivanovii* to metabolize xylose results in the production of blue colonies with a yellow halo. As for *L. monocytogenes*, the colonies produced are blue without halo due to the inability to metabolize xylose. Other *Listeria* sp. that grow on Rapid’ *L. mono* agar will appear as white colonies with or without a yellow halo (Janzten et al., 2006; Zunabovic et al., 2011; Magalhães et al., 2014).

In general, chromogenic media are able to isolate and distinguish *L. monocytogenes* from other *Listeria* sp. and thus allowing a more rapid detection of *L. monocytogenes*. However, the sensitivity and specificity of the culture media may be affected by the types of food matrices (Andritsos et al., 2013). For instance, the study conducted by Aragon-Alegro et al. (2008) indicated that the sensitivity and specificity of CHROMagar^TM^ *Listeria* for the detection of *L. monocytogenes* in sliced cooked ham (56.2% sensitivity and 73.6% specificity), minced beef meat (92.7% sensitivity and 76.8% specificity) and frankfurters (91.2% sensitivity and 84.2% specificity) were different. Hence, there are no particular medium which is perfect for the isolation of *L. monocytogenes* from various food samples (Churchill et al., 2006; Andritsos et al., 2013).

### Cultural Detection and Enumeration of *Listeria monocytogenes*

Traditionally, the detection and identification of pathogens in foods involve the use of culture methods followed by phenotypic confirmation based on standard culture (e.g., haemolysis and phospholipase C), biochemical and immunological identification (Gasanov et al., 2005; Janzten et al., 2006). The conventional methods are simple, sensitive, inexpensive, and important when bacterial culture is required as the end result from positive samples (Churchill et al., 2006; Janzten et al., 2006; Law et al., 2015). Generally, the culture methods involve a two-stage enrichment process followed by plating on a selective differential agar (Beumer and Hazeleger, 2003; Janzten et al., 2006). The procedures may vary depending on the number of cells expected in a sample and/or the official culture reference methods used. The success of culture methods depends on several factors. For instance, the amount and state of the bacteria in the sample, the selectivity of the media (balance between inhibition of competitors and inhibition of the target bacteria), electivity of the isolation medium (difference between the target bacteria and competitive microflora) and the conditions of incubation (e.g., temperature, time, and oxygen; Beumer and Hazeleger, 2003).

The food samples are homogenized prior to the two-stage enrichment process which is divided into pre-enrichment stage and selective enrichment stage that involve incubation for ∼24–72 h at 30–37°C (Churchill et al., 2006; Janzten et al., 2006). Pre-enrichment is carried out in non- or half-selective enrichment medium in order to revive the injured target pathogen and to increase the amount of the target pathogen. In addition, pre-enrichment allows the dilution of inhibitory compounds present in foods such as preservatives and rehydration of bacterial cells sampled from dried or processed food matrices (Gasanov et al., 2005; Dwivedi and Jaykus, 2011; Jadhav et al., 2012; Välimaa et al., 2015). As for selective enrichment, it involves the use of selective medium that will increase the amount of target pathogen while suppress the growth of competing background microflora, thus, enabling the isolation and detection of the target pathogen (Dwivedi and Jaykus, 2011; Välimaa et al., 2015). Selective and differential plating is carried out after the two-stage enrichment process. The analysis is completed if there are no typical colonies can be observed on the selective differential agar and the results are reported as negative. If presumptive positive colonies are isolated, further tests are required to confirm the isolated pathogen such is described below (Jeyaletchumi et al., 2010a; Dwivedi and Jaykus, 2011; Välimaa et al., 2015).

The well-known culture reference methods for the isolation and detection of *L. monocytogenes* in foods are the FDA-BAM, ISO 11290 method, and USDA-FSIS method. These methods are recommended for the detection of *L. monocytogenes* from different food matrices and they utilize different enrichment media as well as plating media. Besides, the incubation time and temperature employed by each culture reference method are slightly different (Gasanov et al., 2005; Churchill et al., 2006; Janzten et al., 2006; Jeyaletchumi et al., 2010a; Välimaa et al., 2015). Numerous researchers have employed these culture reference methods for the investigation of *L. monocytogenes* in foods (Jeyaletchumi et al., 2010a; Goh et al., 2012; Lambertz et al., 2012; Jamali et al., 2013; Kramarenko et al., 2013; Wang et al., 2013; Osman et al., 2014). The culture reference methods are summarized in **Table 1**.

**Table 1:** Summary of each culture reference method for the isolation and detection of *L. monocytogenes* in foods and the detection limit of each method.

Method	Food matrices	Summary of method	Detection limit	Reference
FDA-BAM	Seafood, fruits, vegetables, and dairy products	(1) A 25 g of food sample stomached in 225 mL of BLEB and then incubate at 30°C for 4 h (2) After 4 h of incubation, add selective agents such as acriflavine, nalidixic acid, and cycloheximide into enrichment broth, incubate at 30°C for 48 h (3) Streak enrichment culture onto one of the prescribed selective differential agar plate (Oxford, MOX, or PALCAM) at 24 and 48 h (4) Incubate agar plate at 35°C for 24–48 h (5) Determine the presumptive colonies and then proceed to confirmation of *Listeria* sp. and *L. monocytogenes*	<1 CFU/mL	Hitchins and Jinneman, 2013; Välimaa et al., 2015


ISO 11290-1	All types of foods	(1) For primary enrichment, add *X* g or *X* mL of food sample to 9*X* mL of half Fraser broth, incubate at 30°C for 24 ± 2 h (2) Streak primary enrichment culture onto ALOA and second selective medium (Oxford or PALCAM), incubate at 37°C for 24 ± 2 h. If necessary, further 24 ± 2 h (3) For secondary enrichment, add 0.1 mL of primary enrichment culture to 10 mL of Fraser broth, incubate at 35 or 37°C for 48 ± 2 h (4) Streak secondary enrichment culture onto ALOA plate and second selective medium (Oxford or PALCAM), incubate at 37°C for 24 ± 2 h. If necessary, further 24 ± 2 h (5) Determine the presumptive colonies and then proceed to Confirmation of *Listeria* sp. and *L. monocytogenes*	<1 CFU/g in 25 g	ISO, 2004a; Zunabovic et al., 2011; Välimaa et al., 2015


USDA-FSIS	Red meat, poultry products, and egg products	(1) A 25 g of food sample stomached in 225 mL UVM broth and then incubate at 30 ± 2°C for 20–26 h (2) Streak primary enrichment culture onto MOX plate and then incubate at 35 ± 2°C for 26 ± 2 h. Determine the presumptive colonies from MOX plate. Proceed to confirmation of *Listeria* sp. and *L. monocytogenes* (3) For secondary enrichment, add 0.1 mL of primary enrichment culture to 10 mL of Fraser broth or MOPS-BLEB (4) For Fraser broth, incubate at 35 ± 2°C for 26 ± 2 h. After incubation, observe the broth for the presence of *L. monocytogenes* (darkening of medium due to esculin hydrolysis) (i) If positive, streak 0.1 mL of the Fraser broth onto MOX plate. Incubate MOX plate at 35 ± 2°C for 26 ± 2 h. Determine the presumptive colonies from MOX plate. Proceed to confirmation of *Listeria* sp. and *L. monocytogenes* (ii) If negative, reincubate Fraser broth for further 24 h. Re-examine the Fraser broth for confirmation of darkening. The sample is considered negative for *L. monocytogenes* if no darkening of Fraser broth and no suspected colonies on MOX are observed (5) For MOPS-BLEB, incubate at 35 ± 2°C for 18–24 h (i) After incubation, streak 0.1 mL of the MOPS-BLEB onto MOX plate. Incubate MOX plate at 35 ± 2°C for 26 ± 2 h (ii) Determine the presumptive colonies from MOX plate and proceed to confirmation of *Listeria* sp. and *L. monocytogenes*	<1 CFU/g	USDA-FSIS, 2013; Välimaa et al., 2015

The qualitative information of the pathogen is provided by conventional methods. As for the quantitative information of the pathogen, it is required if the pathogen is detected in the food sample. The enumeration of the level of *L. monocytogenes* contamination in food sample can be done according to the ISO 11290-2 method (ISO, 2004b) and the protocols mentioned in FDA-BAM as well as USDA-FSIS method (**Table 1**). Besides, the FDA and USDA have issued Compliance Policy Guides for food industry regarding the appropriate measures required to control *L. monocytogenes* in food and prevent contamination of food with *L. monocytogenes* (Kraiss, 2008). Enumeration of *L. monocytogenes* in food is important because an initial contamination as few as 1 CFU/100 g *L. monocytogenes* can cause the food unsafe in 32 days, while 10 CFU/g *L. monocytogenes* can cause the food unsafe in 8 days (Salvat and Fravalo, 2004). *L. monocytogenes* is able to grow over a wide range of temperatures, from around -0.4 to 45°C with an optimum temperature of 37°C (International Commission on Microbiological Specifications for Foods [ICMSF], 1996). Hence, this may cause the prevalence of *L. monocytogenes* in food to increase and reach unsafe levels during storage periods or long holding time before retailing. The infectious dose of *L. monocytogenes* for healthy or susceptible individuals has not been established, however, it is estimated to be ∼10^7^–10^9^ CFU in healthy individuals and 10^5^–10^7^ CFU in susceptible individuals such as immunocompromised people or pregnant women (Farber et al., 1996; Smith et al., 2003). In order to enumerate the level of food sample contamination by presumptive *L. monocytogenes*, the primary enrichment broth is quantified prior to incubation, by direct spread plate count on chromogenic media (Hitchins and Jinneman, 2013). If the level of contamination is low, the enumeration of *L. monocytogenes* is done by the most probable number (MPN) technique (Janzten et al., 2006; Jeyaletchumi et al., 2010a; Hitchins and Jinneman, 2013). Besides, some samples may contain particulate material that will interfere with plate count enumeration methods. Hence, MPN technique is applied for these types of samples (Sutton, 2010). MPN technique allows the estimation of population density of viable microorganisms in a sample through replicate liquid broth growth in 10-fold dilutions (Sutton, 2010; Letchumanan et al., 2014). The theoretical basis for MPN technique is to dilute the sample to some extent that inocula in the tubes will occasionally contain viable organisms. A reasonably accurate estimation of the most probable number of cells in the sample can thus be achieved by replicates and dilution series (Sutton, 2010). The FDA-BAM has described 10-fold serial dilution of sample in BLEB with the use of three or more tube MPN culture procedure on each dilution. The samples are incubated at 30°C for 48 h, followed by streaking on selective agar medium (Hitchins and Jinneman, 2013). *L. monocytogenes* can be directly enumerated if chromogenic media is used after MPN enrichment (Janzten et al., 2006; Jeyaletchumi et al., 2010a).

Most probable number technique is more sensitive as compared to direct plating, however, it is more labor intensive and it requires ∼7 days to complete the identification (Janzten et al., 2006; Jeyaletchumi et al., 2010a; Dwivedi and Jaykus, 2011). In MPN technique, the use of selective agar media or chromogenic media may not be selective enough as they may allow the growth of other competitive background microflora, thereby causing difficulties in determining presumptive *L. monocytogenes* (Jeyaletchumi et al., 2010a). MPN technique combined with polymerase chain reaction (PCR) technique is developed in order to overcome these limitations. MPN-PCR technique involves the detection of a particular gene in the target bacteria by PCR instead of isolation of the target bacteria for the enumeration of the bacteria in a sample (Letchumanan et al., 2014). Hence, this technique allows the direct enumeration of *L. monocytogenes* in food without interference of background microflora. The enumeration of *L. monocytogenes* by MPN-PCR technique can be completed in 2 days and this method has higher sensitivity than the standard MPN technique (Jeyaletchumi et al., 2010a). Several researchers have reported the success of MPN-PCR technique for the enumeration of *L. monocytogenes* in various food samples such as fermented sausages (Martín et al., 2004), salad vegetables (Jeyaletchumi et al., 2010b), and raw chicken (Goh et al., 2012).

## Molecular Detection of *Listeria monocytogenes*

The detection of *L. monocytogenes* in food samples by conventional methods is simple, sensitive, and inexpensive if compared with molecular methods (Janzten et al., 2006; Law et al., 2015). However, conventional methods are laborious and time consuming as they require more than a week for the detection and confirmation of pathogen (Dwivedi and Jaykus, 2011; Law et al., 2015; Letchumanan et al., 2015b). Due to the recent advances in molecular technology, molecular methods have been used as an alternative to culture and serological methods for food testing (Gasanov et al., 2005). The detection of a pathogen present in food by nucleic-acid based molecular methods is based on the detection of specific DNA or RNA sequences in the target pathogen. Hence, these genetic methods can provide highly accurate and reliable results as compared to phenotypic methods. Nevertheless, molecular methods require specialized instruments and highly trained personnel (Gasanov et al., 2005; Jadhav et al., 2012; Law et al., 2015). There are various molecular methods available for the detection and identification of *L. monocytogenes*, for instance, PCR, multiplex polymerase chain reaction (mPCR), real-time/quantitative polymerase chain reaction (qPCR), nucleic acid sequence-based amplification (NASBA), loop-mediated isothermal amplification (LAMP), DNA microarray as well as next generation sequencing (NGS) technology.

Polymerase chain reaction has been widely used for the detection of various foodborne bacterial pathogens. This method requires two single-stranded synthetic oligonucleotides or specific primers for the amplification of a specific target DNA sequence in a cyclic three steps process involving the use of a thermal cycler. The PCR amplification products are separated by agarose gel electrophoresis and visualized on the gel as bands with a DNA stain. The specific detection of the genus *Listeria* by PCR involves PCR primers based on the highly conserved 16S rRNA sequence present in all *Listeria* sp. with a resulting 938 bp amplification product (Levin, 2003; Burbano et al., 2006; Goh et al., 2012; Jamali et al., 2013). *L. monocytogenes* can be differentiated from other *Listeria* sp. by exploiting the molecular differences within the PCR amplified 16S rRNA gene, 23S rRNA gene and 16S–23S rRNA intergenic spacer regions (Wang et al., 1992; Graham et al., 1996, 1997; Sallen et al., 1996). In addition, PCR method also detects *L. monocytogenes* at the species level by targeting the virulence genes of the organism (Levin, 2003). Several virulence genes have been identified in *L. monocytogenes* and targeted for the PCR detection of the organism, for example, *hly* (*hlyA*) gene codes for listeriolysin O (LLO; Deneer and Boychuk, 1991; Johnson et al., 1992; Agersborg et al., 1997; Aznar and Alarcón, 2003; Amagliani et al., 2004; Burbano et al., 2006), *iap* gene codes for an invasion-associated protein known as p60 (Agersborg et al., 1997; Aznar and Alarcón, 2003; Swetha et al., 2012), *actA* gene codes for a surface protein known as ActA which is required for intracellular bacterial propulsion and cell to cell invasion (Moriishi et al., 1998; Levin, 2003), *lma*A gene codes for *L. monocytogenes* antigen (lmaA), which also known as *Dth*-18 gene codes for delayed-type hypersensitivity protein (DTH-18 factor; Wernars et al., 1991; Johnson et al., 1992; Levin, 2003), *inlA* gene codes for internalin A (Almeida and Almeida, 2000; Ingianni et al., 2001; Jung et al., 2003), *inlB* gene codes for internalin B (Pangallo et al., 2001; Jung et al., 2003), *prfA* gene codes for positive regulator factor A (PrFA; Simon et al., 1996), *pepC* codes for aminopeptidase C (Winters et al., 1999), *fbp* gene codes for fibronectin-binding protein (Gilot and Content, 2002) and *plcB* Phospholipase C protein (Volokhov et al., 2002). Among these targeted genes, the *hly*A gene is the most frequently chosen target gene for the PCR detection of *L. monocytogenes* (Aznar and Alarcón, 2003; Jadhav et al., 2012). The *hlyA* gene codes for a protein with pore forming activity, which is known as listeriolysin O. This protein is found to be essential for the virulence of *L. monocytogenes* as it is responsible for the lysis of phagocyte vacuole and followed by the escape of *L. monocytogenes* from the vacuole (Kathariou et al., 1987; Cossart et al., 1989; Levin, 2003; Liu, 2006). Besides, it has been discovered that all clinical isolates of *L. monocytogenes* have hemolytic activity due to listeriolysin O and thus the *hlyA* gene is a relevant marker for the identification of *L. monocytogenes* (Groves and Welshimer, 1977; Golsteyn-Thomas et al., 1990).

Other than simple PCR, multiplex PCR (mPCR) is available for a more rapid detection of *L. monocytogenes*. Multiplex PCR is a variant of simple PCR in which multiple gene targets are simultaneously amplified by using several sets of specific primers in a single reaction (Liu et al., 2007). The primer design, concentration of primers, PCR buffer concentrations, quantities of DNA template, Taq DNA polymerase, balance between magnesium chloride and deoxynucleotide concentrations and cycling temperatures are very important for a successful mPCR assay (Markoulatos et al., 2002; Zhao et al., 2014; Law et al., 2015). Multiplex PCR is capable of detecting multiple virulence-associated genes of *L. monocytogenes* in a single PCR mixture. Hence, the possible failure in the detection of virulent *L. monocytogenes* can be prevented (Cooray et al., 1994). In the study conducted by Cooray et al. (1994), *L. monocytogenes* in milk samples was successfully detected by mPCR with primers targeting three virulence-associated genes, *prfA, hlyA*, and *plcB*. Liu et al. (2007) had developed an mPCR assay targeting *inlA*, *inlC*, and *inlJ* genes for the rapid species-specific and virulence-specific determination of *L. monocytogenes*. Besides, mPCR is employed for simultaneous detection of *L. monocytogenes*, *Listeria* sp. and other foodborne pathogens such as *Salmonella* sp., *Escherichia coli* O175:H7, *Vibrio parahaemolyticus*, *Staphylococcus aureus*, *Bacillus cereus* as well as *Campylobacter jejuni* in various food samples (Lawrence and Gilmour, 1994; Gilbert et al., 2003; Jofré et al., 2005; Germini et al., 2009; Kumar et al., 2009; Yuan et al., 2009; Zhang et al., 2009; Zarei et al., 2012; Yang et al., 2013). A novel mPCR assay that can simultaneously detect and discriminate six *Listeria* species including *L. monocytogenes, L. grayi, L. ivanovii, L. innocua, L. welshimeri* and *L. seeligeri* was first developed by Ryu et al. (2013). A rapid mPCR assay for simultaneous detection of *L. monocytogenes*, *S. aureus*, *E. coli* O157:H7, *Salmonella* Enteritidis, and *Shigella flexneri* in meat samples was developed by Chen et al. (2012). Lee et al. (2014) had carried out mPCR assay that can simultaneously detect *L. monocytogenes*, *B. cereus*, *E. coli* O157:H7, *V. parahaemolyticus*, *Salmonella* sp., and *S. aureus* in ready-to-eat food samples. Furthermore, the major *L. monocytogenes* serovars such as 1/2a, 1/2b, 1/2c, and 4b can be differentiated by mPCR targeting marker genes *Imo0730*, *Imo1118*, ORF2819, and ORF2110 (Doumith et al., 2004; Hamdi et al., 2007; Erol and Ayaz, 2011).

The development of real-time or quantitative PCR (qPCR) provides high-throughput analysis and low risk of cross-contamination since post-PCR processing for the detection of PCR products is not required (Fricker et al., 2007). Fluorescent dye such as SYBR green, hydrolysis probe such as TaqMan assays and oligonucleotide hybridization probes such as molecular beacons are used to monitor the PCR products in qPCR (Law et al., 2015). Recently, qPCR is widely used for the detection of foodborne pathogens and multiplex qPCR is also developed for this purpose. This method offers rapid and specific identification as well as quantification of *L. monocytogenes* in a variety of food samples such as soft cheese, fruit juice, fish, vegetables, salads, milk, meat, and crustaceans (Berrada et al., 2006; O’Grady et al., 2008; Kim and Cho, 2010; Garrido et al., 2013; Gianfranceschi et al., 2014). Oravcová et al. (2005) had developed a real-time 5′-nuclease PCR targeting a sequence of the gene *actA* for the identification and quantification of *L. monocytogenes*. In this study, TaqMan probe was used for the detection and quantification of qPCR products. Besides, Barbau-Piednoir et al. (2013) developed a combination of four qualitative SYBRgreen qPCR assays for the detection and discrimination of *Listeria* sp. and *L. monocytogenes* with high accuracy. In these assays, the *iap* and *prs* genes were targeted for detection of *Listeria* sp. and *hlyA* gene was targeted for detection of *L. monocytogenes*. The successful detection of *L. monocytogenes* in fresh produce using molecular beacon-qPCR targeting the *hlyA* gene was first reported by Liming et al. (2004). Furthermore, a novel 5′ exonuclease multiplex qPCR assay for the identification of six *Listeria* sp. including *L. monocytogenes*, *L. seeligeri*, *L. ivanovii*, *L. welshimeri*, *L. grayi*, and *L. innocua* was developed by Hage et al. (2014). In this study, two sets of triplex PCR were designed with one set identifying *L. seeligeri*, *L. welshimeri*, and *L. monocytogenes* and another set identifying *L. ivanovii*, *L. grayi*, and *L. innocua*. The *Listeria* species were differentiated by targeting their respective species-specific target genes and TaqMan probe was used to monitor the multiplex qPCR products.

Additionally, commercial qPCR kits for the detection of *L. monocytogenes* are available and this allows laboratories in food industry to adapt qPCR testing easily (Janzten et al., 2006). The examples of these commercial qPCR kits include BAX^®^ System Real-time PCR Assay *Listeria monocytogenes* (DuPont-Qualicon), Probelia^®^ *Listeria monocytogenes* PCR System (Bio-Rad), LightCycler^®^ *Listeria monocytogenes* Detection Kit (Roche/Biotecon), TaqMan^®^ *Listeria monocytogenes* Detection Kit (Applied Biosystems), GeneVision^®^ Rapid Pathogen Detection System for *Listeria monocytogenes* (Warnex), ADIAFOOD rapid pathogen detection system for *Listeria monocytogenes* (AES Chemunex), CycleavePCR^®^ *Listeria monocytogenes* (*inlA* gene) Detection Kit (TaKaRa Bio, Inc.) and iQ-Check *L. monocytogenes* kit (Bio-Rad Laboratories; Liming et al., 2004; Rodríguez-Lázaro et al., 2004; Becker et al., 2005; Janzten et al., 2006; Liu et al., 2012).

There is no doubt that PCR-based detection methods are rapid, highly sensitive, and specific. However, these methods require thermocycling system. Alternative methods have been developed for the amplification of nucleic acids under isothermal conditions. Two of the most commonly used isothermal nucleic acid amplification methods for the detection of foodborne pathogens are LAMP and NASBA.

Several types of LAMP assays such as multiplex LAMP, real-time LAMP, *in situ* LAMP and reverse-transcription LAMP have been developed and utilized for the detection of foodborne pathogens (Ye et al., 2011; Law et al., 2015). Studies have shown that LAMP assay has high specificity and it exhibits higher sensitivity than PCR assays in the detection of *L. monocytogenes*. For example, Tang et al. (2011) conducted a sensitive and specific LAMP assay for the detection of *L. monocytogenes* with primers that target the *hlyA* gene region. In this study, the LAMP assay was evaluated against conventional PCR method for the detection of *L. monocytogenes* in food. The results indicated that LAMP assay was 100 times more sensitive than the conventional PCR assay. Besides, a real-time quantitative LAMP that amplifies the *hlyA* gene of *L. monocytogenes* was designed by Shan et al. (2012). This LAMP assay was then used to detect *L. monocytogenes* in four different types of retail food samples such as raw meat, vegetables, deli, and seafood. The study also proven that LAMP assay was more sensitive than PCR in the detection of *L. monocytogenes*. A double LAMP (dLAMP) assay was first conducted by Wu et al. (2014) for the detection of *L. monocytogenes* in food samples including pork, beef, chicken, mutton, shrimp, fish, and quick-frozen rice flour products. LAMP primers targeting the *hlyA* and *iap* genes of *L. monocytogenes* were used to ensure the dLAMP assay is more rapid, sensitive and specific. The results of this study showed that dLAMP assay was more sensitive and less time consuming as compared to normal LAMP assay. Recently, LAMP has been commercialized as kits for the detection of *L. monocytogenes*, for instance, Loopamp^®^ *Listeria monocytogenes* Detection Kit (Eiken Chemical, Co., Ltd.) and Isothermal Master Mix (OptiGene; Wang et al., 2015).

In general, NASBA often involves in the amplification of mRNA targets under isothermal conditions (Leone et al., 1998). NASBA selectively amplifies the mRNA targets even in the presence of genomic DNA and it has been used to detect various foodborne pathogens (Simpkins et al., 2000). The main advantage of NASBA over other molecular detection methods is its ability to detect viable bacterial cells that are present in environmental samples and food samples (Simpkins et al., 2000; Cook, 2003). A highly specific NASBA system was developed by Blais et al. (1997) for the detection of *L. monocytogenes* with primers targeting the *hlyA* mRNA sequences. The NASBA system was capable of detecting low numbers of *L. monocytogenes* (<10 CFU/g) in artificially contaminated dairy and egg products after 48 h enrichment period. Nevertheless, false-positive results were reported and the researchers suggested that the reason for this could be due to cross-contamination of NASBA reactions with amplicons from previous amplifications performed at the same site. The post-NASBA product detection steps involving agarose gel electrophoresis, enzyme-linked gel assay, enzymatic bead-based detection and numerous probing and/or blotting techniques can be laborious. Hence, homogenous real-time NASBA that utilizes fluorescently labeled probes (e.g., molecular beacon) to monitor the amplicons is developed in order to overcome this problem (Leone et al., 1998). A molecular beacon-based real time NASBA assay for the detection of *L. monocytogenes* in cooked ham and smoked salmon slices was first described by Nadal et al. (2007). Sequence from the mRNA transcript of *hly* gene was used as a target for this assay and the detection limit of this assay for *L. monocytogenes* was 400 CFU/mL. This study also involved the use of a commercial NASBA kit, which was NucliSens^®^ Basic Kit (bioMérieux) for the detection of *L. monocytogenes*.

DNA microarrays, which were initially being applied for the study of gene expression, could be used for the investigation of microbial evolution and epidemiology as well as for the detection of foodborne pathogens (Gasanov et al., 2005; Severgnini et al., 2011). DNA microarrays comprise multiple specific oligonucleotide probes (with sequence length ranges from 25 to 80 bp) or PCR probes which are coated on to glass slides or chips. The target nucleic acid which can be either DNA, mRNA or cDNA is labeled with fluorescent dye and then applied to the DNA microarray. The target nucleic acid will bind to its corresponding oligonucleotide probe and the hybridization is detected by production of fluorescent signal from probe-sample complex. DNA microarrays are capable of detecting multiple foodborne pathogens simultaneously and thus suitable for high-throughput analysis (De Boer and López, 2012; Law et al., 2015). An oligonucleotide DNA microarray assay that can simultaneously detect and discriminate six *Listeria* sp. including *L. monocytogenes*, *L. ivanovii*, *L. innocua*, *L. seeligeri*, *L. grayi*, and *L. welshimeri* was performed by Volokhov et al. (2002). The microarray assay was based on *iap*, *hly*, *inlB*, *plcA*, *plcB*, and *clpE* genes for the identification of *Listeria* species. Oligonucleotide DNA microarray was also used for the simultaneous detection of different foodborne bacterial pathogens which include *L. monocytogenes*, *E. coli* O157:H7, *Salmonella enterica* and *C. jejuni* in food samples (Suo et al., 2010). DNA microarrays provide high-throughput analysis but the shortcoming is that large amounts of target DNA or RNA are needed for these methods (Gasanov et al., 2005).

The majority of bacterial genome sequences available today have been generated using the Sanger chain termination sequencing chemistries. Despite being very instrumental in the rise of the field of genomics, it is time consuming as well as resource intensive (Sanger et al., 1977; Medini et al., 2008). The post-Sanger era sequencing technologies, the NGS technologies, have been developed since 2005 to permit extremely rapid high-throughput whole genome sequencing (WGS) hence providing a broader application of comparative genomics (Medini et al., 2008; Shendure and Ji, 2008; Letchumanan et al., 2015a). Due to rapid decreasing costs for sequencers and reagents, a bacterial genome sequence can be obtained within a few days for less than US$500 (Didelot et al., 2012), and more than 36,000 bacterial genome sequences are available in public databases (Reddy et al., 2015). Other than serving as a detection tool, WGS is also a feasible tool for retrospective epidemiological analyses and is frequently used for the latter purpose. Genome sequencing of several *L. monocytogenes* strains have revealed serotype- and strain-specific characteristics of *L. monocytogenes* (Orsi et al., 2008; Fretz et al., 2010; Gilmour et al., 2010) and provided novel insights into the genomic causes underlying pathogenicity and survival in food and food processing settings (Buchrieser and Glaser, 2011).

A listeriosis outbreak in Oklahoma, USA in the year 1988 was linked to the consumption of turkey franks contaminated with *L. monocytogenes* produced in a food processing facility in Texas, USA (Centers for Disease Control, and Prevention [CDC], 1989). In 2000, 11 states in the US faced listeriosis outbreak affecting 29 individuals including four fatalities, and it was linked to consumption of deli turkey meat produced in the same facility (Stone and Shoenberger, 2001; Olsen et al., 2005). Using NGS, Orsi et al. (2008) revealed that the human listeriosis outbreak in 2000 in the USA was caused by a *L. monocytogenes* strain that persisted in that food processing plant for over 12 years in which the same strain has also been responsible for a sporadic case in 1988.

In 2008, *L. monocytogenes* serotype 1/2a caused an outbreak of listeriosis associated with ready to eat meat products in Canada, resulting in 22 deaths and at least 57 illnesses (Gilmour et al., 2010). The authors reported the first real-time application of WGS during an active listeriosis outbreak investigation using the high-throughput to characterize two outbreak-associated isolates of *L. monocytogenes*. In 2009 and 2010, another large listeriosis outbreak occurred in Austria, Germany, and the Czech Republic due to intake of a traditional Austrian cheese called “Quargel,” an acid curd cheese with a red smear made from skimmed pasteurized milk (Fretz et al., 2010). Molecular typing via PFGE revealed that two different *L. monocytogenes* strains, both serotype 1/2a (Pichler et al., 2011). From June 2009 to January 2010 Quargel outbreak clone 1 (QOC1) was the culprit in 14 cases, including five with a lethal outcome (Fretz et al., 2010). Whereas between December 2009 and February 2010, Quargel outbreak clone 2 (QOC2) accounted for 20 cases, which resulted in three deaths (Fretz et al., 2010). Rychli et al. (2014) sequenced and analyzed the genomes of both outbreak strains in order to retrospectively investigate the extent of genetic diversity between the two strains. WGS analysis revealed that these two strains have distinct *in vitro* virulence potential despite originating from similar serovar (Rychli et al., 2014).

The development of benchtop sequencers using NGS technology such as 454 or GS FLX^TM^ (Roche), MiSeq (Illumina) and Ion Torrent Personal Genome Sequencer (PGM; Life Technologies) will enable bacterial WGS even in small research and clinical laboratories (Didelot et al., 2012). WGS has already been actively used for the characterization of bacterial isolates in several large outbreaks in the world (Gilmour et al., 2010; Reuter et al., 2013) and also being used as a tool for retrospective epidemiological analyses (Orsi et al., 2008; Rychli et al., 2014). In the near future this technology is likely to substitute currently used typing methodologies due to its ultimate resolution and sensitivity (Sabat et al., 2013). However, NGS also has its limitation. Until now, it is still too laborious and time-consuming to obtain useful data for routine surveillance (Fournier et al., 2014). Library preparation and sequencing protocol requires adept and skillful technician; however, this limitations are likely to be overcome due to higher level of automation which will lead to a more streamline processed. Bioinformatics analysis and interpretation, as well as computational hardware are also another challenges to be solved especially by small laboratories (Fournier et al., 2014). In addition, a fundamentally unsolved question is how the sequences should be examined for epidemiological characterization (Sabat et al., 2013). More examples of studies that involved the application of molecular methods for the detection and identification of *L. monocytogenes* in food samples are listed in **Table 2**.

**Table 2:** Application of molecular methods for the detection and identification of *L. monocytogenes* in various food samples.

Detection method	Gene target	Food matrix	Reference
Simple PCR	*hlyA, iap*	Naturally contaminated fish samples	Swetha et al., 2012
	*hlyA*	Naturally contaminated raw meat (chicken, beef, and fish), milk and milk products (raw milk, cheese, and curd)	Khan et al., 2013
	*actA*	Artificially contaminated milk, pork, and water	Zhou and Jiao, 2005
Multiplex PCR	*iap,hly*	Artificially contaminated milk	Zeng et al., 2006
	*plcA*, *hlyA*, *actA*, *iap*	Artificially contaminated milk	Rawool et al., 2007
	16S rRNA, *iap*	Naturally contaminated deli meat samples: pork and chicken products	Liu et al., 2015
Real-time/quantitative PCR	*prfA*	Artificially contaminated raw milk, salmon, pâté, and green-veined cheese Naturally contaminated fish, meat, meat products, and dairy products	Rossmanith et al., 2006


	*Iap*	Artificially contaminated milk	Hein et al., 2001
	*Hly*	Artificially contaminated pork meat	Gattuso et al., 2014
	16S-23S rRNA intergenic spacer regions	Artificially contaminated soft cheese, fermented sausage, cured ham, and ready-to-eat salad Naturally contaminated fresh meat, fresh sausages, fermented sausages, fresh cheeses, and ripened cheeses	Rantsiou et al., 2008
	16S rRNA	Leafy vegetables: collard green, cabbage, lettuce, mixed parsley, chinese cabbage, spring onion bunches, spinach, wild chicory, arugula, and watercress	De Oliveira et al., 2010
	*inlA*	Artificially contaminated chicken meat	Navas et al., 2006
LAMP	*prfA*	Artificially contaminated milk	Cho et al., 2014
	*hlyA*	Artificially contaminated chicken, pork, ground beef, and milk powder	Wan et al., 2012
	*iap*	Artificially and naturally contaminated raw milk	Wang et al., 2011
NASBA	16S rRNA	Artificially contaminated chicken breast meat, soft cheese, shrimps, dry sausage, minced meat (pork and beef), radish and mushrooms	Uyttendaele et al., 1995
DNA microarray	Genomic DNA of *L. monocytogenes*	Artificially contaminated milk	Bang et al., 2013
NGS	Whole genome of *L. monocytogenes*	Deli turkey meat Ready to eat meat Quargel cheese	Orsi et al., 2008 Gilmour et al., 2010 Rychli et al., 2014

There are many advantages of using molecular methods for the detection and identification of *L. monocytogenes*. For instance, the main advantages of molecular detection methods are due to their high sensitivity and specificity. Nonetheless, limitations can be found in these methods such as some molecular methods can be costly, complex and require trained personnel. The advantages and limitations of molecular methods are summarized and listed in **Table 3**.

**Table 3:** Advantages and limitations of molecular methods for the detection and identification of *L. monocytogenes.*

Molecular methods	Advantages	Limitations	Reference
Simple PCR	• High sensitivity and specificity • Accurate and reliable results	• Sensitivity may be affected by non-optimized protocols and PCR inhibitors • Requires DNA purification step	Mandal et al., 2011; Letchumanan et al., 2014; Law et al., 2015
Multiplex PCR	• High sensitivity and specificity • Enables simultaneous detection of multiple foodborne pathogens	• Sensitivity may affected by non-optimized protocols and PCR inhibitors • Primer design and other mPCR conditions (e.g., primer concentration, PCR buffer concentration, and quantities of DNA template) are important	Markoulatos et al., 2002; Mandal et al., 2011; Law et al., 2015
Real-time/quantitative PCR	• Higher sensitivity and specificity than simple PCR • More rapid than simple PCR and mPCR as post-amplification products processing is not required • Assay can be multiplexed • Allows high-throughput analysis	• Costly • Sensitivity may affected by PCR inhibitors • Trained personnel is needed	Oravcová et al., 2005; Mandal et al., 2011; Letchumanan et al., 2014; Law et al., 2015
LAMP	• Higher sensitivity and specificity than PCR • Cost-effective • Simple • Operates without thermal cycling system	• Complicated primer design	Letchumanan et al., 2014; Zhao et al., 2014; Law et al., 2015
NASBA	• Sensitive and specific • Cost-effective • Operates without thermal cycling system • Enables the detection of viable bacteria	• Requires viable bacteria • Might not be easy to handle RNA	Lauri and Mariani, 2009; Zhao et al., 2014; Law et al., 2015
DNA microarray	• High sensitivity and specificity • Enables simultaneous detection of multiple foodborne pathogens • Allows high-throughput analysis	• Costly • Trained personnel is needed • Requires large amount of target DNA or RNA	Gasanov et al., 2005; Lauri and Mariani, 2009; Law et al., 2015
NGS	• High sensitivity and specificity • Enables simultaneous detection of multiple foodborne pathogens • Allows high-throughput analysis • Enable the analysis of whole genome of the pathogens	• Costly • Trained personnel is needed • Requires Bioinformatics skills for analysis and interpretation • Computationally intensive	Sabat et al., 2013; Fournier et al., 2014

## Conclusion

Early detection of *L. monocytogenes* contaminated food is crucial as it can prevent the outbreaks of foodborne illness. Till date, the culture reference methods mentioned in this review are still applicable and being used in many studies. Simple PCR and mPCR have been used routinely for rapid, sensitive, and specific screening as well as confirmation of *L. monocytogenes*. The introduction of LAMP and NASBA allow accurate and cost-effective screening of *L. monocytogenes*. For large number of samples, high-throughput assays such as qPCR and DNA microarray are often used for the detection of *L. monocytogenes*. Cultural and molecular techniques are continuously being developed and improved in order to provide higher sensitivity and specificity of *L. monocytogenes* detection. The advancements of molecular methods allow the rapid detection of *L. monocytogenes* in food samples with high sensitivity and specificity whilst substituting the lengthy and laborious conventional detection methods. Molecular methods have provide many advantages, nonetheless, there are still some limitations in these methods such as the need to use highly advance technology that are costly compared to conventional methods. The combined use of two or more detection methods is also possible and may improve the accuracy of detecting *L. monocytogenes*. Still, there are great potential for the development and application of new techniques for foodborne pathogens detection and analysis.

## Conflict of Interest Statement

The authors declare that the research was conducted in the absence of any commercial or financial relationships that could be construed as a potential conflict of interest.
